# Screening and Referral Care Delivery Services and Unmet Health-Related Social Needs: A Systematic Review

**DOI:** 10.5888/pcd18.200569

**Published:** 2021-08-12

**Authors:** Emily Ruiz Escobar, Shweta Pathak, Carrie M. Blanchard

**Affiliations:** 1 University of North Carolina Eshelman School of Pharmacy, Chapel Hill, North Carolina

## Abstract

**Introduction:**

Unmet health-related social needs contribute to high patient morbidity and poor population health. A potential solution to improve population health includes the adoption of care delivery models that alleviate unmet needs through screening, referral, and tracking of patients in health care settings, yet the overall impact of such models has remained unexplored. This review addresses an existing gap in the literature regarding the effectiveness of these models and assesses their overall impact on outcomes related to experience of care, population health, and costs.

**Methods:**

In March 2020, we searched for peer-reviewed articles published in PubMed over the past 10 years. Studies were included if they 1) used a screening tool for identifying unmet health-related social needs in a health care setting, 2) referred patients with positive screens to appropriate resources for addressing identified unmet health-related social needs, and 3) reported any outcomes related to patient experience of care, population health, or cost.

**Results:**

Of 1,821 articles identified, 35 met the inclusion criteria. All but 1 study demonstrated a tendency toward high risk of bias. Improved outcomes related to experience of care (eg, change in social needs, patient satisfaction, n = 34), population health (eg, diet quality, blood cholesterol levels, n = 7), and cost (eg, program costs, cost-effectiveness, n = 3) were reported. In some studies (n = 5), improved outcomes were found among participants who received direct referrals or additional assistance with indirect referrals compared with those who received indirect referrals only.

**Conclusion:**

Effective collaborations between health care organizations and community-based organizations are essential to facilitate necessary patient connection to resources for addressing their unmet needs. Although evidence indicated a positive influence of screening and referral programs on outcomes related to experience of care and population health, no definitive conclusions can be made on overall impact because of the potentially high risk of bias in the included studies.

**Summary**

**What is already known on this topic?**

Little is known about the overall impact of screening and referral programs that address unmet health-related social needs on outcomes related to experience of care, population health, and cost.

**What is added by this report?**

Although screening and referral programs positively affected outcomes related to experience of care and population health, definitive conclusions about their overall impact could not be determined.

**What are the implications for public health practice?**

This study synthesizes evidence to inform health care administrators and policy makers considering the expansion of screening and referral programs to address unmet health-related social needs.

## Introduction

Up to 80% of health outcomes can be attributed to social determinants of health (SDOH), the conditions in which we grow, live, and work (1,2). Adverse SDOH include food insecurity, housing instability, unemployment, and other unmet health-related social needs (3), which often contribute to negative health outcomes, including an increased risk for diabetes, hypertension, and heart disease (4–7). Recently, higher unemployment rates and changes in health insurance coverage due to the ongoing COVID-19 pandemic have further compromised health care access and increased the number of people with unmet needs (8,9).

**Figure 1:**
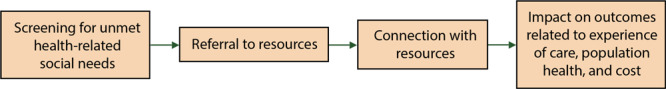
Processes and potential impact on outcomes of screening and referral-based delivery services for addressing unmet health-related social needs among patients in a healthcare setting.

Health care organizations (HCOs) offer a natural setting for integration of clinical care, public health, and community-based services (10,11). The Centers for Medicare and Medicaid Services (CMS) has recognized the potential value in leveraging the infrastructure of HCOs for addressing health-related social needs. As part of the Accountable Health Communities initiative, CMS provides incentives for HCOs to consider solutions that address unmet needs by potentially improving population health and reducing system costs to drive overall performance (12). One common approach to the screening and referral–based care delivery model includes the identification of unmet needs through a screening questionnaire, followed by a referral component that addresses or mitigates unmet needs through referrals to appropriate resources, and subsequently evaluates the impact of this screening and referral program (12–14) (Figure 1).

Although implementation of such screening and referral-based programs has increased in recent years (14), we found no review that summarized evidence on the impact of these programs on care outcomes. Therefore, in accordance with PRISMA (Preferred Reporting Items for Systematic Reviews and Meta-Analyses) guidelines (15), we answered the following population, intervention, comparison, and outcomes question (PICO): What is the impact of screening and referral programs targeting unmet health-related social needs in health care settings on outcomes related to experience of care, population health, and costs?

## Methods

### Data sources

Because CMS only started implementing screening and referral–based care delivery models in 2016 (12), we searched PubMed to identify relevant peer-reviewed articles published over the past 10 years as of March 2020 to capture results from any pilot and demonstration projects before and after this time frame. Search terms were derived with the help of a subject librarian and included the following terms: (“social determinants of health” OR “social determinants” OR “social needs” OR food insecurity OR housing OR transportation OR employment) AND (screening OR needs assessment OR test) AND (referrals OR collaboration OR address needs) AND (“primary care” OR primary health care OR health services) NOT (biological OR psychology OR mental health). Our search terms did not contain an exhaustive list of all social determinants described in the literature. Specific health-related social needs (eg, food insecurity, housing) included in the search indicate the needs commonly addressed by current screening and referral programs. Additionally, we scanned the bibliographies of all articles that met the inclusion criteria and other literature reviews (16,17). To maximize our final article yield, older studies published before January 1, 2010, obtained from bibliographies, were included if they met the inclusion criteria.

### Study selection

Articles were included if they were written in English and described an intervention in a health care setting that 1) used a screening tool to identify unmet health-related social needs, 2) referred screened patients with positive results (or positive screens) to resources offering assistance (eg, on-site provision of food or referral to a food bank), and 3) reported any care outcomes resulting from the screening and referral components described in 1) and 2), beginning with program recruitment or referral uptake. After the study selection, all outcomes were categorized into experience of care, population health, and cost-related based on the Institute for Healthcare Improvement (IHI) Triple Aim framework (18), which targets 3 dimensions for optimizing performance in HCOs: 1) improving the patient experience of care through quality and satisfaction; 2) improving health of the patient population, and 3) reducing the per capita cost of care.

Using the Triple Aim framework as a guideline, outcomes related to the patient *experience of care* included outcomes resulting from the referral (eg, patient use of resource) and patient-reported outcomes (eg, self-reported changes in social needs, patient satisfaction with the screening and referral intervention). Outcomes related to *population health* describe any changes in indicators pertaining to patient health (eg, blood pressure trends, diet intake). *Cost-related outcomes* included any changes in health care costs, utilization, or cost-effectiveness evaluation.

Articles were excluded if the intervention was in a non–health care setting (eg, community settings such as food banks), if the care delivery services focused solely on individual behavior-related determinants (eg, smoking, physical inactivity, alcohol consumption) rather than social determinants, or if the program did not include a screening and/or referral component. Articles were also excluded if we could not ascertain whether on-site screening for health-related social needs was performed or if solely process-related, descriptive screening outcomes (eg, number of screenings, number of referrals) were reported.

Screening of titles and abstracts was carried out by 2 reviewers (E.R.E., S.P.) using Microsoft Excel (Microsoft Corporation). Once relevant articles were independently identified, each reviewer completed a full-text review of the selected articles. We planned to resolve discrepancies during the article selection process by using consensus among the authors (E.R.E., S.P., C.B.), but no discrepancies occurred.

### Data extraction

From each eligible article, we extracted the following: author name(s), year of publication, place of origin, health care setting(s), target population, study design, sample size, screening tool used, targeted unmet health-related social need(s), referral approach, referral site, outcome(s) assessed, and study results.

### Risk of bias assessment and data analysis

Valid and complementary assessment tools for randomized (19) and nonrandomized studies (20) were used to examine risk of bias. For randomized clinical trials, we used the Cochrane tool (19) to make critical assessments (low risk, high risk, and unclear risk) of included studies in 6 domains: sequence generation, allocation concealment, blinding, incomplete outcome data, selective outcome reporting, and “other sources of bias.” For nonrandomized studies, we made similar critical assessments (low risk, high risk, and unclear risk) using the RoBANS tool (Risk of Bias Assessment for Nonrandomized Studies) (20) for a slightly different set of 6 domains: selection of participants, confounding variables, measurement of exposure, blinding of outcomes assessment, incomplete outcome data, and selective outcome reporting. For both randomized and nonrandomized studies, the final assessment within and across studies was based on the responses to individual domains.

A qualitative synthesis of results across studies was performed. Meta-analysis was not performed because of heterogeneity in the study populations, interventions, and outcomes of included studies.

## Results

**Figure 2:**
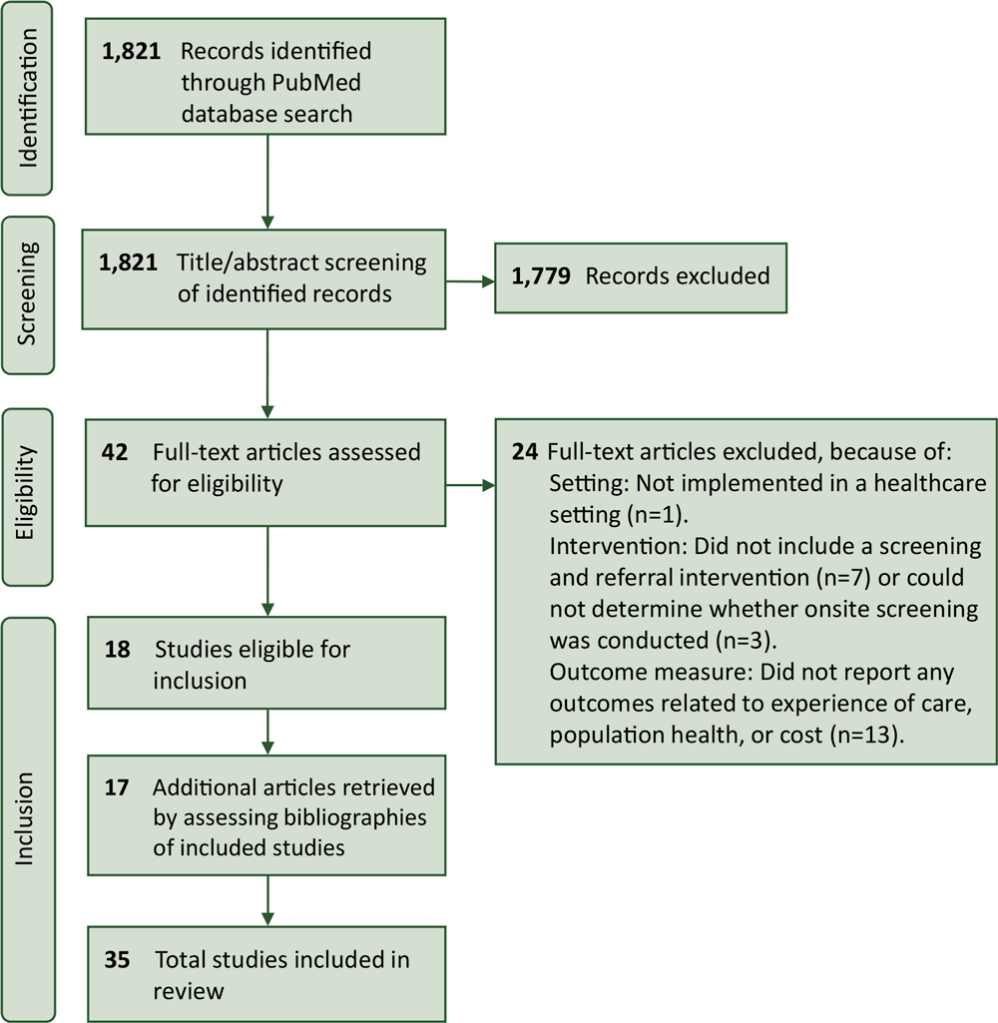
PRISMA (Preferred Reported Items for Systematic Reviews and Meta-Analysis) diagram for identification of included studies.

A total of 1,821 articles were identified from the PubMed database search (Figure 2). After applying the PICO question and our inclusion criteria, 42 articles were selected for full-text review, of which 18 met the inclusion criteria. An additional 17 articles were included from bibliographies, bringing the total to 35 articles in the final review. Seven (20%) studies were randomized control trials, 6 (17%) were observational studies that compared outcomes within the intervention group to a nonintervention comparison group, and the rest examined outcomes within an intervention group only (n = 22; 63%).

### Risk of bias assessment

Randomized studies demonstrated a potentially high risk (n = 6) or unclear risk of bias (n = 1) (Table 1). Insufficient or lack of information about blinding of participants, personnel, or outcomes indicated that potential selection, performance, and detection biases were present. Additionally, all nonrandomized studies (n = 28) were assessed as having a potentially high risk of bias (Table 2). The most common domains demonstrating high risk were blinding of outcomes assessment (n = 28), confounding variables (n = 19), and participant selection (n = 13).

**Table 1:** Risk of Bias Assessment for Included Randomized Studies, Screening and Referral to Identify Unmet Health-Related Social Needs in Health Care Settings

Author, Year (Reference)	Risk of Bias Assessment						
	Random Sequence Generation	Allocation Concealment	Blinding, Participants and Personnel/ Outcomes	Incomplete Outcomes Data	Selective Reporting	Other Sources of Bias	Overall Assessment
Dubowitz H, 2009 (21)	Low	Unclear^a^	High/High	Low	Low	Low	High
Ferrer RL, 2019 (22)	Low	Low	High/High	Low	Low	High	High
Garg A, 2015 (23)	Low	Unclear^a^	Low/Low	Low	Low	Low	Unclear
Gottlieb LM, 2016 (24)	Low	High	High/High	Low	Low	Low	High
Haas JS, 2015 (25)	Low	Unclear^a^	High/Low	Low	Low	Low	High
Sege R, 2015 (26)	Low	Unclear^a^	High/High	Low	Low	High	High
Silverstein M, 2004 (27)	Low	Unclear^a^	Low/High	Low	Low	Low	High

a Insufficient information provided to determine whether allocation concealment was performed.

**Table 2:** Risk of Bias Assessment for Included Nonrandomized Studies, Screening and Referral to Identify Unmet Health-Related Social Needs in Health Care Settings

Author, Year (Reference)	Risk of Bias Assessment						
	Participant Selection	Confounding Variables	Measurement of Exposure (Referral Service)	Blinding of Outcome Assessments	Incomplete Outcomes/ Loss to Follow-up	Selective Reporting	Overall Assessment
Aiyer JN, 2019 (28)	Low	High	Low	High	Low	Low	High
Beck AF, 2012 (29)	High	High	Low	High	Low	Low	High
Beck AF, 2014 (30)	High	High	Low	High	Low	Low	High
Berkowitz SA, 2017 (31)	Low	Low	Low	High	Low	Low	High
Coker AL, 2012 (32)	Low	Low	Low	High	Low	Low	High
Dicker RA, 2009 (33)	Low	High	Low	High	Low	Low	High
Dubowitz H, 2012 (34)	Low	Low	Low	High	Low	Low	High
Fiori KP, 2020 (35)	Low	Low	Low	High	Low	Low	High
Fleegler EW, 2007 (36)	High	Low	Low	High	Low	Low	High
Fox CK, 2016 (37)	Low	High	Low	High	Low	Low	High
Garg A, 2010 (38)	Low	High	Low	High	Low	Low	High
Garg A, 2012 (39)	Low	High	Low	High	Low	Low	High
Hassan A, 2015 (40)	Low	High	Low	High	Low	Low	High
Hsu C, 2019 (41)	High	High	Low	High	Low	Low	High
Juillard C, 2015 (42)	High	High	Low	High	Low	Low	High
Klein MD, 2013 (43)	Low	High	Low	High	Low	Low	High
Krasnoff M, 2002 (44)	Low	High	Low	High	Low	Low	High
Marpadga S, 2019 (45)	High	High	Low	High	Low	Low	High
Morales ME, 2016 (46)	High	Low	Low	High	Low	Low	High
Palakshappa D, 2017 (47)	High	Low	Low	High	High	Low	High
Patel MR, 2018 (48)	High	High	Low	High	Low	Low	High
Pettignano R, 2011 (49)	High	High	Low	High	Low	Low	High
Power-Hays A, 2020 (50)	High	High	Low	High	Low	Low	High
Schickedanz A, 2019 (51)	High	Low	Low	High	Low	Low	High
Smith R, 2013 (52)	High	High	Low	High	Low	Low	High
Smith S, 2016 (53)	Low	High	Low	High	Low	Low	High
Stenmark SH, 2018 (54)	Low	High	Low	High	Low	Low	High
Uwemedimo OT, 2018 (55)	Low	Low	Low	High	Low	Low	High

### Settings, populations, and unmet health-related social needs

All included studies (n = 35) had a screening and referral component and originated in the US (Tables 1 and 2). Most screening and referral programs were implemented in pediatric clinics (n = 15) (21,26,29,30,34–37,39,43,47,49,50,54,55) and other primary care practices (n = 11) (22,23,25,27,28,31,32,38,40,41,53); the rest (n = 9) were in other settings (24,33,42,44–46,48,51,52). Included studies defined target populations by health conditions or behavioral risk factors (eg, patients with diabetes, or patients who smoke), and/or demographic characteristics (eg, age, sex).

The social needs addressed included education (eg, poor literacy, health education) (23,24,27,33,38–41,49–52,55), unemployment and income insecurity (eg, vocational training, financial burden) (23,24,26,31,33,36,38–41,43,48–52,55), food insecurity (22–24,26,28,30,31,35–41,43,45–47,49–51,53–55), housing insecurity (eg, poor housing conditions, homelessness) (23,24,26,29,31,35,36,38–41,43,49–52,55), interpersonal safety (eg, intimate partner violence) (21,24,32–36,38,39,43,44,49,55), transportation to health care site (24,31,35,39,41,50,51,55), and others (eg, counseling needs, childcare/eldercare services, access to services) (21,23,24,31–36,38–41,43,49–52,55) (Table 3). Although some programs (n = 13) addressed a single unmet social need (22,27–30,37,44–48,53,54), more than half (n = 21) addressed multiple needs (21,23–26,31–36,38–41,43,49–52,55). One study (42) was a cost-effectiveness analysis of a screening and referral program addressing multiple needs (52).

**Table 3:** Characteristics of Included Studies, Screening and Referral to Identify Unmet Health-Related Social Needs in Health Care Settings

Author, Year; Location (Reference)	Setting; Target Population	Screening Tool and Targeted Unmet Health-Related Social Need	Referral Approach; Referral Site	Study Design, Sample Size^a^	Outcome Assessed	Summary of Results
**Experience of care outcomes**						
Smith S, 2016; San Diego, CA (53)	Setting: 3 student-run free clinics. Population: Adults (aged >18 y)	USDA US Household Food Security Survey 30-day version, targeted food insecurity	Approach: Indirect referral^b^ with on-site assistance.^c^ Site: Local food pantries, monthly on-site food distributions, and on-site same-day SNAP enrollment	Cross-sectional study, 1-group design (n = 430)	Experience of care (referral uptake^d^)	15% (66 of 430) of total patients used a food pantry. 15% (64 of 430) enrolled in SNAP. 48% (208 of 430) of screened patients had diabetes, of whom 97% (201 of 208) received on-site monthly food boxes
Fox CK, 2016; Minnesota (37)	Setting: 1 pediatric weight management clinic. Population: Households with children	Hunger Vital Sign, targeted food insecurity	Approach: Direct referral.^e^ Site: Food bank (Second Harvest Heartland) offered on-site assistance with SNAP application	Prospective pilot study, 1-group design (n = 116)	Experience of care (referral uptake^d^)	8% (3 of 40) of eligible patients completed SNAP enrollment process.
Palakshappa D, 2017; Pennsylvania (47)	Setting: 6 pediatric clinics. Population: Households with children	Hunger Vital Sign in EHR, targeted food insecurity	Approach: Direct referral.^e^ Site: Nonprofit organization (Benefits Data Trust) assisted with applications to government benefits	Prospective mixed-methods study, 1-group design (n = 4,371)	Experience of care (referral uptake^d^)	26% (32 of 122) of patients with food insecurity consented to a direct referral. 3% (1 of 32) of patients enrolled in SNAP.
Stenmark SH, 2018; Colorado (54)	Setting: 2 pediatric clinics. Population: Households with children	Hunger Vital Sign, targeted food insecurity	Approach: Indirect referral^b^ evolved into direct referral.^e^ Site: Nonprofit organization (Hunger Free Colorado) offered assistance with applications to federal and community resources	Descriptive, prospective study, 1-group design, number of screened patients not provided; 1,586 patients were referred	Experience of care (referral uptake^d^)	Connection rate between patients and referral site increased from 5% to 75% after the program moved from indirect to direct referral. 6% (100 of 1,586) of patients enrolled in SNAP.
Marpadga S, 2019; San Francisco, CA (45)	Setting: 1 diabetes clinic. Population: Patients with diabetes	Hunger Vital Sign, targeted food insecurity	Approach: Indirect referral^b^ with on-site assistance.^c^ Site: Multiple, including programs that offered free groceries, on-site prepared meals, home-delivered meals, and medically tailored meals (Project Open Hand)	Qualitative study; semistructured interviews, 1-group design (n = 240)	Experience of care (referral uptake^d^)	13% (31 of 240) of screened patients were interviewed. 32% (10 of 31) of participants connected with food resources: 3% (1 patient) with a program providing free groceries and 29% (9 patients) with a program providing medically tailored meals.
Beck AF, 2012; Cincinnati, OH (29)	Setting: 1 pediatric primary care clinic. Population: Households with children	EHR-based screening, targeted poor housing conditions	Approach: Warm handoff.^f^ Site: On-site medical–legal partnership offered help with legal housing problems	Descriptive, retrospective study, 1-group design, number of screened patients not provided, 16 caregivers referred	Experience of care (referral uptake^d^)	71% (10 of 14) of referred housing units with outcome data resulted in housing condition repairs. 58% (11 of 19) of building complexes with the same owner received substantial systemic repairs.
Silverstein M, 2004; Seattle, WA (27)	Setting: 4 health clinics. Population: Low-income households with children	Program-developed tool, targeted education	Approach: *Intervention:* Direct referral.^e^ *Control*: Indirect referral.^b^ Site: US Department of Health and Human Services program (Head Start)	Randomized controlled trial, intervention (n = 123) vs control (n = 123)	Experience of care (referral uptake^d^)	Intervention group had more children who connected with the education resource (41%, 50 of 123 vs 18%, 22 of 123; adjusted difference, 17%; 95% CI, 8%–27%) and more children who actively attended the program (25%, 31 of 123 vs 11%, 14 of 123; adjusted difference, 12%; 95% CI, 3%–21%) than the control group.
Dicker RA, 2009; San Francisco, CA (33)	Setting: 1 level I trauma center. Population: Patients aged between 12–30	Screening tool (not specified) targeted risk of reinjury	Approach: Warm handoff^f^. Site: Case management services, including help with court advocacy, driver’s license, educational resources, vocational training, mental health and drug treatment, and more	Program evaluation study, 1-group design, number of screened patients not provided, 44 enrolled	Experience of care (referral uptake^d^)	23% of patients with a positive screen for unmet health-related social needs (45 of 195) received full case management services including help with court advocacy, education, vocational training, mental health/drug treatment, employment needs, housing needs, and receiving a driver’s license.
Coker AL, 2012; Unknown location (32)	Setting: 6 primary care clinics. Population: Women (aged >18 years)	Program-developed tool (56) targeted intimate partner violence	Approach: *Intervention*: Indirect referral,^b^ warm handoff,^f^ and on-site assistance.^c^ *Control:* Indirect referral.^b^ Site: Multiple, including coalition services, safety planning, and on-site counseling and support (intervention group only)	Quasi-experimental, longitudinal cohort study, intervention (n = 138) vs control (n = 93)	Experience of care (referral uptake^d^)	A similar number of women reported using the referral resource in the intervention and control group (21.4% vs 17.4%; *P* = .43). More intervention women connected with the on-site advocate (32.8% vs 4.4%; *P* < .001) and had lower IPV scores and fewer depressive symptoms (*P* = .07; *P* = .01) than the control.
Klein MD, 2013; Cincinnati, OH (43)	Setting: 3 pediatric clinics. Population: Households with children	EHR-based screening (57) targeted income, child food insecurity, poor housing conditions, domestic violence, parental depression, and anhedonia	Approach: Warm handoff.^f^ Site: On-site medical–legal partnership offered help with legal problems	Descriptive cohort study, number of enrolled participants not provided, 1-group design; 1,614 patients referred	Experience of care (referral uptake^d^)	1,617 legal cases were pursued by 1,614 referred families. 90% (1,742 of 1,945) of legal outcomes were positive, including improvements in housing conditions, public benefits, education, or provision of legal advice. 10% (n = 203) related to either inability to reconnect with the family or issue resolution.
Uwemedimo OT, 2018; Queens, NY (55)	Setting: 1 hospital-based pediatric practice. Population: Households with children (<18 y)	FAMNEEDS targeted parent counseling and education needs, food insecurity, housing/utility insecurity, interpersonal safety, transportation, unemployment	Approach: Warm handoff^f^ before indirect referral.^b^ Site: Unspecified partner CBOs	Pre-post intervention study, 1-group design (n = 148)	Experience of care (referral uptake^d^)	31% (46 of 148) of households reported using the program-provided resources at 12-month follow-up. More limited English proficiency caregivers used resources (38.4% vs 18.4%, *P* = .03) than English-proficient caregivers, and more noncitizen caregivers used referrals (37.4% vs 23.1%, *P* = .04) than US citizens.
Garg A, 2015; Boston, MA (23)	Setting: 8 community health centers. Population: Households with infants (<6 mo)	Program-developed tool targeted parent education needs, childcare needs, food insecurity, housing insecurity, unemployment	Approach: *Intervention*: Indirect referral^b^ with on-site assistance.^c^ *Control*: Indirect referral.^b^ Site: Unspecified CBOs	Randomized controlled trial, intervention (n = 168) vs control (n = 168)	Experience of care (referral uptake^d^)	Intervention mothers were more likely to enroll in a new community resource (39% vs 24%; aOR = 2.1; 95% CI, 1.2–3.7), had greater odds of being employed or enrolled in a job training program (aOR = 44.4; 95% CI, 9.8–201.4), receiving childcare support (aOR = 6.3; 95% CI, 1.5–26.0), fuel assistance (aOR = 11.9; 95% CI, 1.7–82.9), and lower odds of being in a homeless shelter (aOR = 0.2; 95% CI, 0.1–0.9) than mothers in control group.
Fiori KP, 2020; Bronx, NY (35)	Setting: 1 pediatric clinic. Population: Households with children	EHR-based Health Leads–adapted tool targeted poor access to health care, childcare and eldercare needs, food insecurity, housing insecurity, interpersonal safety, legal needs, transportation	Approach: Warm handoff.^f^ Site: Unspecified CBOs	Pragmatic prospective cohort study, 1-group design (n = 4,948)	Experience of care (referral uptake^d^)	43% (123 of 287) of patients referred to a community health worker had “successful” referrals. These patients either accessed, obtained, or used the recommended community-based service or support.
Pettignano R, 2011; Atlanta, GA (49)	Setting: 1 pediatric clinic. Population: Households with children with sickle cell disease	Screening tool (not specified) targeted legal needs associated with child needs (eg, childcare, child abuse), education, health insurance, interpersonal safety, unemployment, food insecurity, housing insecurity, and income insecurity	Approach: Warm handoff^f^ to HeLP program with on-site assistance.^c^ Site: On-site medical–legal partnership offered help with legal problems	Descriptive, retrospective cohort study, number of enrolled participants not provided, 1-group design, 69 patients referred	Experience of care (referral uptake^d^)	106 legal cases were pursued by 69 referred households. 93% (n = 99) of the cases were closed. 21% (21 of 99) of the closed cases resulted in measurable gain of benefits including obtaining food stamps, Social Security insurance, family stability, employment, and/or housing and education benefits.
Garg A, 2012; Baltimore, MD (39)	Setting: 1 pediatric clinic. Population: Households with children	Health Leads targeted education needs, food insecurity, health insurance, housing insecurity, income insecurity, interpersonal safety, transportation needs, unemployment	Approach: Indirect referral.^b^ Site: Unspecified CBOs	Prospective cohort study, 1-group design (n = 1,059)	Experience of care (referral uptake^d^)	50% (530 of 1,059) of families enrolled in at least 1 community-based resource within 6 months of accessing the on-site Health Leads desk.
Power-Hays A, 2020; Boston, MA (50)	Setting: 1 pediatric hematology clinic. Population: Patients with sickle cell disease	The WE CARE app targeted childcare needs, educational needs, food insecurity, housing insecurity, income insecurity, transportation needs, unemployment	Approach: Indirect referral^b^ or warm handoff.^f^ Site: Unspecified local CBOs	Qualitative quality improvement project, 1-group design (n = 132)	Experience of care (referral uptake^d^)	45% (42 of 92) of patients who were referred and available for follow-up reported reaching out to the CBO.
Hassan A, 2015; Boston, MA (40)	Setting: 1 adolescent/young adult clinic. Population: Patients aged 15–25	Program-developed tool targeted access to health care, education needs, food insecurity, housing insecurity, income insecurity, fitness and safety equipment needs, unemployment	Approach: Indirect referral.^b^ Site: Unspecified CBOs	Prospective interventional study, 1-group design (n = 401)	Experience of care (referral uptake^d^, patient-reported outcomes)	40% (104 of 259) of patients with a positive screen contacted the referral site of which 50% (52 of 104) had their problem resolved. 60% (155 of 259) did not contact the referral site but 45% (70 of 155) reported having resolved their problem.
Krasnoff M, 2002; Unknown location (44)	Setting: 1 level I trauma center. Population: Women aged 18–65	Partner Violence Screen (58) targeted IPV	Approach: Warm handoff.^f^ Site: On-site case manager and other unspecified community-based resources	Observational case study, 1-group design (n = 528)	Experience of care (referral uptake^d^, patient-reported outcomes)	84% (475 of 562) of women with a positive screen consented to meeting with an on-site advocate, of whom 54% (258 of 475) then agreed to meet with a case manager. At follow-up, 24% (127 of the 528) of women reported they no longer believed they were at risk for violence from their abuser.
Haas JS, 2015; Boston, MA (25)	Setting(s): 13 primary care clinics. Population: Adults that smoke	Web-based referral system HelpSteps targeted multiple social needs	Approach: *Intervention*: Direct referral^e^ before indirect referral.^b^ *Control*: No referral. Site: External specialist (direct referral), unspecified CBOs (indirect referral), and provision of free NRT patches	Randomized clinical trial, intervention (n = 399) vs control (n = 308)	Experience of care (referral uptake^d^, patient-reported outcomes)	68.7% (274 of 399) of intervention participants connected with the external tobacco treatment specialist, while 20.1% reported using the HelpSteps referral. Intervention participants who connected with the specialist (21.2% vs 10.4%; *P* = .009) or used the HelpSteps referral (43.6% vs 15.3%; *P* < .001) were more likely to quit than those who did not.
Hsu C, 2019; San Pablo, CA (41)	Setting: 1 primary care practice. Population: Adults	Health Leads targeted childcare needs, food insecurity, health literacy, housing insecurity, income insecurity, transportation	Approach: Warm handoff.^f^ Site: Unspecified community-based resources	Qualitative study; semistructured interviews, 1-group design (n = 102)	Experience of care (referral uptake, patient-reported outcomes)	Patients reported concrete changes in their lives including healthier diets, decreased stress or worry, and increased feeling of stability; some reported as resolved immediate food, transportation, or health care needs, and others reported physical or mental/emotional benefits.
Fleegler EW, 2007; Boston, MA (36)	Setting: 2 pediatric clinics. Population: Households with children aged 0–6	Program-developed tool targeted poor access to health care, food insecurity, housing insecurity, income insecurity, and intimate partner violence	Approach: Indirect referral^b^ Site: Unspecified local agencies	Cross-sectional descriptive study, 1-group design (n = 450)	Experience of care (referral uptake^d^, patient satisfaction)	63% (73 of 115) of referrals received by 79 households led to contact with the referral agency. 82% (60 of the 73) of households considered their referral sites helpful.
Garg A, 2010; Baltimore, MD (38)	Setting: 1 medical home. Population: Households with children	WE CARE-based tool targeted child needs (eg, after-school programs, childcare, child school failure), education needs, food insecurity, health insurance, housing insecurity, public benefits needs, income insecurity, IPV, unemployment, safety equipment, and other (eg, smoking, drug or alcohol abuse)	Approach: Warm handoff.^f^ Site: Unspecified CBOs	Longitudinal cohort pilot study, 1-group design (n = 59)	Experience of care (referral uptake^d^, patient satisfaction)	32% (19 of 59) of parents that used the on-site Help Desk reported enrolling in at least 1 community program. 21% (4 of the 19) enrolled in ≥2 community programs. More than 90% of parents who enrolled in a community resource were very or somewhat satisfied.
Gottlieb LM, 2016; San Francisco and Oakland, CA (24)	Setting: 2 safety-net hospitals. Population: Households with children	14-item questionnaire targeted needs related to childcare, education, food insecurity, health insurance, housing insecurity, income insecurity, interpersonal safety, legal aid, transportation, unemployment	Approach: *Intervention:* Warm handoff.^f^ *Control:* Indirect referral.^b^ Site: Unspecified community, hospital, and government-based resources	Randomized clinical trial, intervention (n = 872) vs control (n = 937)	Experience of care (patient-reported outcomes)	At 4-months postenrollment, intervention participants reported fewer unmet social needs (mean change of −0.39 vs 0.22; *P* < .001) and greater improvement in their child’s health than control participants (mean change of −0.36 vs 0.12; *P* < .001).
Dubowitz H, 2009; Baltimore, MD (21)	Setting: 1 pediatric clinic. Population: Households with children aged 0–5	Parent Screening Questionnaire (59) targeted child maltreatment risk factors including parental depression, parental substance abuse, harsh punishment, major parental stress	Approach: *Intervention:* Indirect referral^b^ and warm handoff,^f^ if needed. *Control:* No referral. Site: Multiple, including local community resources and on-site social workers	Randomized controlled trial, intervention (n = 308) vs control (n = 250)	Experience of care (patient-reported outcomes)	Postintervention, the intervention group had fewer families that filed child protective services reports (13.3% vs 19.2%; *P* = .03), and fewer instances of possible medical neglect including nonadherence (4.6% vs 8.4%; *P* = .05) and delayed immunizations (3.3% vs 9.6%; *P* = .002) than the control group. Control group had more parent-reported harsh punishment (*P* = .04).
Dubowitz H, 2012; Maryland (34)	Setting: 18 pediatric practices. Population: Mothers with children	Parent Screening Questionnaire (59) targeted child maltreatment risk factors including parental depression, parental substance abuse, harsh punishment, major parental stress	Approach: *Intervention:* Indirect referral^b^ and warm handoff^f^ if needed. *Control*: No referral. Site: Multiple, including local community resources and on-site social workers	Case-control study; intervention (n = 595) vs control (n = 524)	Experience of care (patient-reported outcomes)	Intervention mothers reported less psychological aggression initially and 12 months later (initial effect size *P* = .006; 12-month effect size *P* = .047) and fewer minor physical assaults (initial effect size *P* = .02; 12-month effect size *P* = .04) than control.
**Population health outcomes**						
Beck AF, 2014, Cincinnati, OH (30)	Setting: 1 pediatric clinic. Population: Households with infant(s) aged <12 months	Hunger Vital Sign targeted food insecurity	Approach: *Recipients*: Indirect referral^b^ and on-site assistance.^c^ *Nonrecipients*: No referral. Site: Unspecified CBOs and on-site provision of formula cans	Prospective, difference-in-difference study, recipients (n = 1,042) vs nonrecipients (n = 4,029)	Experience of care (referral uptake^d^), Health	Experience of care: All recipients were more likely to have been referred to social work (29.2% vs 17.6%; *P* < .001), or the medical–legal partnership (14.8% vs 5.7%; *P* < .001) than nonrecipients. Health: By 14 months, recipients versus nonrecipients were more likely to have completed a lead test and developmental screen (both *P* < .001), and a full set of well-infant visits (42% vs 28.7%; *P* < .001).
Sege R, 2015; Boston, MA (26)	Setting: 1 hospital-based pediatric clinic. Population: Households with newborn aged <10 weeks	Screening tool (not specified) targeted food insecurity, housing insecurity, income insecurity	Approach: *Intervention*: Warm handoff.^f^ *Control*: No referral. Site: On-site medical–legal partnership	Randomized controlled trial, intervention (n = 167) vs control (n = 163)	Experience of care (referral uptake^d^), Health	Experience of care: Intervention versus control showed accelerated access to resources (baseline, 2.8% vs 1.6%; 6 months, 3.2% vs 2.7%; 12 months, 3.7% vs 3.2%; *P* = .03). Health: Intervention versus control group had more infants that completed their 6-month immunization schedule by age 7 and 8 months (77% vs 63%; *P* < .005 and 88% vs 78%; *P* < .01, respectively), more likely to have ≥5 routine preventive care visits by age 1 year (78% vs 67%; *P* < .01), and less likely to have visited the emergency department by age 6 months (37% vs 50%; *P* = .021).
Patel MR, 2018; Michigan (48)	Setting: 1 endocrinology clinic. Population: Patients with diabetes	Program-developed tool targeted financial burdens	Approach: Indirect referral.^b^ Site: Unspecified local and national resources for financial burden and disease management	1-group pre–post pilot study (n = 104)	Experience of care (referral uptake^d^, patient satisfaction), Health	Experience of care: More participants were using low-cost resources at 2-month follow-up compared with baseline, such as online diabetes education (40% vs 29%; *P* = .05) and assistance programs related to blood glucose supplies (40% vs 16%; *P* = .03). Participants found the resource tool highly acceptable across 15 indicators (eg, 93% “learned a lot,” 98% “topics relevant”). Health: Fewer patients reported skipping doses of medicines due to cost concerns (4% vs 11%; *P* = .03) compared with baseline.
Smith R, 2013; San Francisco, CA (52)	Setting: 1 hospital. Population: Victims of violent trauma aged 10–30 years	Screening tool (not specified) targeted high risk for reinjury and others, including need for court advocacy, driver’s license, education, employment, family counseling, housing, mental health, vocational/professional training, substance abuse help	Approach: Warm handoff.^f^ Site: Unspecified risk-reduction resources	Retrospective cohort study, 1-group design (n = 141)	Experience of care (referral uptake^d^), Health	Experience of care: For 6 years of the program, 254 clients received on-site case management services; a total of 617 needs were identified. 70% (430 of 617) of identified needs were met. Health: The violent injury recidivism rate dropped from an initial 16% to 4.5% by the end of the program.
Berkowitz SA, 2017; Boston, MA (31)	Setting: 3 primary care practices. Population: Adults with chronic disease	Health Leads targeted access to medications, elder care needs, food insecurity, housing insecurity, income insecurity, transportation needs, unemployment	Approach: *Participants:* Warm handoff.^f^ *Nonparticipants*: No referral. Site: Unspecified CBOs and public benefits	Pragmatic difference-in-difference evaluation study, participants (n = 1,021) vs nonparticipants (n = 301)	Experience of care (referral uptake^d^), Health	Experience of care: 58% (1,021 of 1,774) of patients with a positive screen enrolled in the program and connected with the on-site advocate. 29.7% of reported needs were closed as “successful,” 27.9% as “equipped,” 34.9% as “unsuccessful,” and 7.1% were handled with a rapid resource referral. Health: Participants versus nonparticipants demonstrated greater improvement in blood pressure (SBP differential change −1.2; 95% CI, −2.1 to −0.4; DBP differential change −1.0; 95% CI, −1.5 to −0.5), and LDL-C (differential change −3.7; 95% CI, −6.7 to −0.6), but no change in HbA_1c_ (differential change −0.04%; 95% CI, −0.17% to 0.10%).
Morales ME, 2016; Chelsea, MA (46)	Setting: 1 obstetric clinic. Population: Women	Program-developed tool targeted food insecurity	Approach: *Recipients:* Indirect referral^b^ and on-site assistance.^c^ *Nonrecipients:* No referral. Site: Food for Families program, which included referral to local food pantries and on-site support with SNAP or WIC enrollment	Retrospective cohort study, 2-group design, recipients (n = 145) vs nonrecipients (n = 145)	Experience of care (referral uptake^d^), Health	Experience of care: 67% (97 of 145) of women referred to the program enrolled. Health: Recipients demonstrated better blood pressure trends during pregnancy (SBP 0.2015 mm Hg/wk lower; *P* = .006 and DBP 0.1049 mm Hg/wk lower; *P* = .02). No blood pressure trend among nonrecipients, and no differences in blood glucose trends between the 2 groups (*P* = .40).
Ferrer RL, 2019; San Antonio, TX (22)	Setting: 1 primary care clinic. Population: Patients with type 2 diabetes	Hunger Vital Sign targeted food insecurity	Approach: *Intervention*: Warm handoff.^f^ *Control*: Indirect referral.^b^ Site: Regional food bank	Randomized controlled trial, intervention (n = 19) vs control (n = 24)	Experience of care (patient-reported outcomes), Health	Experience of care: Intervention group received an average of 7.8 food allotments and were visited at home by a community health worker an average of 2.6 times. Health: Intervention versus control demonstrated a greater drop in HbA_1c_ levels (mean difference of −3.09 vs −1.66; *P* = .01), improved STC-Diet scale (mean differences of 2.47 vs 0.06; *P* = .001), but no significant BMI difference (mean differences of –0.17 vs 0.84; *P* = .43).
**Cost-related outcomes**						
Aiyer JN, 2019; North Pasadena, TX (28)	Setting: 1 federally qualified health center and 2 school-based clinics. Population: Households with children	Hunger Vital Sign targeted food insecurity	Approach: Indirect referral.^b^ Site: Food prescription to local food pantry	1-group design, pre–post mixed methods evaluation study, n = 242	Experience of care (referral uptake^d^, patient-reported outcomes), Cost-related (program costs)	Experience of care: 71.1% (172 of 242) of referred patients redeemed their prescription at the food pantry. 94.1% (162 of 172) participants reported a decrease in the prevalence of their food insecurity. Cost-related: Program costs was $12.20 per participant per prescription redemption.
Schickedanz A, 2019; Southern CA (51)	Setting: 1 health care system. Population: Predicted high-utilizer patients	Health Leads targeted child-related needs, educational needs, food insecurity, housing insecurity, income insecurity, transportation needs, unemployment	Approach: *Intervention*: Indirect referral.^b^ *Control*: No referral. Site: Multiple community-based resources including food banks, housing programs, and other agencies	Prospective difference-in-difference study, intervention (n = 7,107) vs control (n = 27,118)	Experience of care (referral uptake^d^), Cost-related (utilization)	Experience of care: 53% (1,984 of 3,721) of screened participants reported social needs, but only 10% of those connected with resources. Cost-related: Intervention versus control showed 2.2% decline in utilization visits (*P* = .058) over 1-year postintervention, including emergency department visits, inpatient hospitalizations, and ambulatory visits. Greater declines in total utilization for all low-socioeconomic status subgroups in intervention versus control (*P* < .001).
Juillard C, 2015; San Francisco, CA (42)	Refer to Smith R, 2013 (52)	Refer to Smith R, 2013 (52)	Refer to Smith R, 2013 (52)	Cost-effectiveness analysis of Smith R, 2013 (52)	Cost-related (cost effectiveness and cost savings)	Cost-related: Realized substantial health benefits (24 QALYs) and savings ($4,100) if implemented for 100 people.

Abbreviations: BMI, body mass index; CBO, community-based organization; DBP, diastolic blood pressure in mm Hg; EHR, electronic health record; HbA_1c_, hemoglobin A_1c_; FAMNEEDS, Family Needs Screening Program; HeLP Program, Health Law Partnership; IPV, intimate partner violence; KIND, Keeping Infants Nourished and Developing; LDL-C, low-density lipoprotein cholesterol in mg/dL; NRT, nicotine replacement therapy; QALYs, quality-adjusted life years; SBP, systolic blood pressure in mmHg; SNAP, Supplemental Nutrition Assistance Program; USDA US HFSS, US Department of Agriculture US Household Food Security Survey; WIC, Special Supplemental Nutrition Program for Women, Infants, and Children.

a Reported as the total number of participants who underwent screening. If the study did not report number of screenings, the number of referrals was reported as the sample size.

b A referral approach in which health care providers simply hand over information about relevant referral sites to the patient (eg, a list of local food banks and their contact information).

c Additional on-site services may include assistance with applying to community-based resources or connection to other resources through a helpdesk, and/or on-site provision of supplies.

d Refers to participants who connected to necessary resources expressed as a percentage or ratio of all participants who had a positive screen or those who consented to a referral.

e A referral approach that requires the patient’s consent to forward their contact information to the corresponding internal or external resource. The referral site then directly contacts the patient.

f A referral approach in which patients are introduced to an on-site intermediary person in the health care organization (eg, community health worker, case manager) who works to connect them to referral sites.

### Screening component

The programs described in the included studies employed various screening tools (eg, the Hunger Vital Sign [https://childrenshealthwatch.org/public-policy/hunger-vital-sign/], Health Leads [https://healthleadsusa.org/]) to identify unmet need(s). Most studies (n = 19) (21,22,25,28,30,31,34,35,37–39,41,44,45,47,50,51,53,54) either used tools that had been previously validated in existing literature (60,61) or used tools developed in-house (n = 11) (23,24,27,29,32,36,40,43,46,48,55). Other studies (n = 4) (26,33,49,52) did not specify a screening tool.

Screening assessments were facilitated by clinic staff (n = 9) (21,28,34,35,37,41,46,50,54), health care providers (n = 6) (23,30,31,39,47,49), and others (n = 7) (33,38,43,45,46,50,55). Some assessments (n = 4) were administered to patients online (25,27,36,40).

### Referral component

Studies featured HCOs that partnered with various community-based organizations (CBOs) or expanded their internal resources to include assistance programs that addressed immediate unmet needs. Five studies reported on providing on-site social assistance services including CBO eligibility applications (32,45,49,53) and distribution of food supplies (30,53). Although descriptions of community collaboration were sparse, referral sites included CBOs such as food banks, nutrition programs, intimate partner violence agencies, housing programs, and early childhood education programs.

Additionally, we found 3 referral approaches: indirect, direct, and warm handoff. In an *indirect referral*, health care providers simply hand over information about the referral site(s) to the patient (eg, distribute a list of local food banks and their contact information to patients who have a positive screen for food insecurity). A *direct referral* approach is when the HCO directly forwards a patient’s contact information to a referral site contingent on the patient’s consent and is often administered through health information exchange tools. The referral site then follows up with the patient to assist in the patient’s application or enrollment in programs to alleviate unmet needs. A *warm handoff* is when an on-site intermediary person in the HCO (eg, community health worker, social worker) assists patients who have a positive screen with connecting to the referral site.

Indirect referrals and warm handoffs were the most common referral approaches (n = 29) reported (21–24,26,28–36,38–41,43–46,48–53,55). The rest (n = 5) were studies that reported direct referrals (25,27,37,47,54). Studies with 2 groups either compared outcomes in the intervention group to a control group that received no referral (n = 8) (21,25,26,30,31,34,46,51) or to a control group that received a different type of referral (n = 5) (22–24,27,32).

### Qualitative synthesis of outcomes

Most studies (n = 25) (21,23–25,27,29,32–41,43–45,47,49,50,53–55) reported only outcomes related to experience of care (Table 3), which included referral uptake (ie, participants who connected with or used necessary resources expressed as a percentage or ratio of all participants who had a positive screen or consented to a referral) and patient-reported outcomes (ie, self-reported changes in social needs, diet, health, and patient satisfaction). Other studies reported population health outcomes (n = 7) (22,26,30,31,46,48,52), which included changes in indicators of patient health such as systolic and diastolic blood pressure, glycosylated hemoglobin levels (HbA_1c_), body mass index (BMI), low-density lipoprotein cholesterol, medication adherence, appointment adherence, violent injury recidivism rates, and preventive care outcomes (ie, completing lead tests, developmental screens, infant immunization schedules, or well-infant visit sets). Only 3 studies reported cost-related outcomes (28,42,51), including evaluation of program costs, changes in health care utilization, or cost-effectiveness.

### Experience of care outcomes

**Referral uptake.** Data on participants or participating families who connected with or used necessary resources were expressed either as a percentage of all participants who had a positive screen or as a percentage of those who consented to/received a referral (referral uptake). Although most studies (n = 30) reported some information on patient connection to the referral site, the reported results were highly contextual and varied widely from study to study. For example, some studies reported connection rates as low as 3% (47) while others reported rates as high as 75% (54).

**Patient-reported outcomes**. Nine studies (21,24,28,36,38,40,41,44,48) reported positive findings on patient-reported outcomes. For example, 1 study interviewing patients with unmet needs (41) reported participants being able to make concrete changes in their lives as a result of screening and referral, including resolving immediate social needs, a healthier diet, or physical and mental/emotional benefits; another study (28) found that participants’ self-reported food insecurity decreased by 94.1%.

Three studies (36,38,48) that investigated patient satisfaction reported positive feedback on referral sites and program tools. More than 80% of patients found their referral sites helpful in 1 study (36), and more than 90% of parents enrolled in a community-based resource reported being “very” or “somewhat” satisfied in a different study (38). Similarly, participants with diabetes in another study reported high acceptability across multiple survey items in their program’s resource tool (eg, 93% “learned a lot,” 98% found “topics relevant”) (48).

### Health outcomes

Seven (22,26,30,31,46,48,52) studies that examined outcomes related to population health found some positive changes in health indicators. After addressing income insecurity, 7% fewer patients (*P* = .03) reported skipping doses of medicines because of financial concerns (48). Another study found a drop in the violent injury recidivism rate from an initial 16% to 4.5% by the end of the program (52). Other studies found improved preventive care outcomes, including faster completion of lead tests, developmental screens, infant immunization schedules among participants (77% vs 63% completed by age 7 months, *P* = .002 and 88% vs 78% by age 8 months, *P* = .008) (26), and greater likelihood of completing a full set of well-infant visits by 14 months (42% vs 28.7%; *P* < .001) (30). Changes reported in intervention participants enrolled in screening and referral programs compared with those who did not receive a referral included improvements in systolic blood pressure and diastolic blood pressure trends during pregnancy (46) (*P* = .004), and a modest differential change in systolic blood pressure of −1.2 mm Hg (95% CI, −2.1 to −0.4), diastolic blood pressure of −1.0 mm Hg (95% CI, −1.5 to −0.5), and improved low-density lipoprotein cholesterol (differential change −3.7 mg/dL; 95% CI, −6.7 to −0.6) among participants with diabetes (31).

### Cost-related outcomes

Only 3 studies examined cost-related outcomes. One study targeting food insecurity with a food prescription program (28) found program costs to be $12.20 per person per redemption. Additionally, participants reported an average $57 savings per week on grocery bills. Another study targeting multiple social needs (51) reported a decreased likelihood of health care utilization among the intervention group compared with the control group. Also, a cost-effectiveness study (42) of a hospital-based violent injury prevention intervention (52) yielded 25.6 quality-adjusted life years (QALYs, a standardized measure of disease burden in cost-effectiveness evaluations that typically combines both survival and health-related quality of life to guide decisions on the distribution of limited health care resources) versus 25.3 for the non violent injury prevention group, with net costs of $5,892 per patient versus $5,923 for the non violent injury prevention group.

### Impact of referral approach on all outcomes

Studies comparing direct (27,54) to indirect referral reported greater referral uptakes with direct referral. One study (27) found that intervention participants receiving direct referrals reported a greater percentage of children who connected with an education resource (41% vs 18%) and actively attended the development program (25% vs 11%) than intervention participants who received an indirect referral. Another study (54) reported patient connection to referral sites increasing from 5% to 75% when the approach was changed from indirect to direct referral.

Some studies compared indirect referrals paired with additional services (eg, on-site assistance) in the intervention to a control group that received indirect referrals only. One such study (32) found a similar percentage of participants using the referral resource (21.4% vs 17.4%; *P* = .43) as the control group. However, a greater percentage of intervention participants than control participants connected with the on-site advocate (32.8% vs 4.4%; *P* < .001). Similarly, another study (23) reported that intervention participants had greater odds than control participants of being employed or enrolled in a job training program (aOR = 44.4), receiving childcare support (aOR = 6.3) and fuel assistance (aOR = 11.9), and lower odds of being in a homeless shelter (aOR = 0.2).

Two studies (22,24) compared outcomes in patients receiving a warm handoff with patients who received an indirect referral. The intervention group receiving warm handoff (22) had decreased HbA_1c_ levels (mean differences of −3.09 vs −1.66; *P* = .012), improved STC (Starting the Conversation)-Diet scale (62) (mean differences of 2.47 vs 0.06; *P* = .001), but no difference in BMI (mean differences of –0.17 vs 0.84; *P* = .43) compared with control participants. Similarly, intervention participants in another study (24) reported fewer unmet social needs (mean change of −0.39 vs 0.22; *P* < .001) and greater improvement in their child’s health than control participants (mean change of −0.36 vs 0.12; *P* < .001).

## Discussion

The body of evidence on the relationship between unmet health-related social needs and poor patient outcomes has continued to grow in recent years. In response, screening and referral programs have expanded to mitigate unmet health-related social needs among patients in health care settings (12,14).

This review found 35 studies on screening and referral delivery services that reported outcomes related to patients’ experience of care, population health, and cost. The delivery service targeted patients with different chronic conditions and demographic characteristics, aiming to mitigate different health-related unmet needs.

We found some indication that screening and referral programs had a generally positive impact on outcomes related to experience of care, population health, and cost. Patient connection to referral sites and patient-reported outcomes such as self-reported diet intake, resolution of unmet health-related social needs, overall well-being, and patient satisfaction increased. Indicators of health such as blood pressure trends, low-density lipoprotein cholesterol, and medication adherence improved. Additionally, results indicated an improvement in QALYs, decreased likelihood of health care utilization, and modest savings associated with these programs.

Overall, included studies revealed a high risk of bias for elements related to study design and evaluation. Thus, we were unable to draw any definitive conclusions about the impact of screening and delivery services on any outcome.

The linkage of patients to resources seemed to be influenced by the type of referral and the degree of navigation within the HCOs and collaboration between the HCOs and the CBOs involved. Results suggested that patients were more successful in connecting with resources when partnered CBOs are more directly involved (ie, direct referral), or if referral efforts were made through on-site intermediaries such as community health workers to direct patients in contacting or applying to referral sites (ie, warm handoff).

Studies have indicated (37,41,47) that referral uptake was influenced by accessibility to referral sites, including patient eligibility criteria and intensity of time and labor required to access resources. For instance, the application process for SNAP (Supplemental Nutrition Assistance Program) is lengthy and complex (63). Such barriers, as speculated in the literature (64), can explain why, despite participating in screening and referral programs, patients can have difficulties in accessing some resources. In short, the degree of referral uptake can be subject to various program characteristics including referral approach, on-site assistance, and accessibility of referral sites.

This review serves as a call to action for policy makers, advocates, and care providers to facilitate screening and referral delivery services through strong collaborations among health care, public health, and community sectors to address unmet health-related and social needs. Such programs can offer a comprehensive solution for health care administrators and insurers looking to improve the health of their patient population, reduce system costs, and optimize overall performance by addressing social determinants of health in their patient populations and delivering high-quality person-centered care. Research with stronger study designs and rigorous evaluation methodologies is needed to establish a strong evidence base of the effectiveness of screening and referral delivery services. Future studies can further explore social-needs screening in mental/behavioral health settings that target individual behavior-related determinants of health (eg, smoking, alcohol abuse) along with social determinants.

To our knowledge, this review is the first study to provide an overview of the impact of screening and referral programs on outcomes related to experience of patient care, population health, and costs. Although our search for articles was performed in accordance with PRISMA guidelines for a systematic review (15), our study was exploratory. We limited our search to peer-reviewed articles and 1 database, which might have excluded other results reported in the gray literature or in other databases.

In summary, literature on the impact of screening and referral programs in HCOs had a tendency toward high risk of bias. Although the evidence indicated promising changes in patient connection to resources, patient-reported outcomes, patient satisfaction, and some health indicators, no definitive conclusions could be made about the impact of such programs on outcomes related to experience of care, population health, and cost. This study can inform public health professionals, administrators, and policy makers about the impact of implementing screening and referral care delivery services in health care settings, paving the way for the expansion of such programs to improve population health.
